# Medical Fees, Etc.

**Published:** 1880-03

**Authors:** 


MEDICAL FEES, ETC.—In almost all communities a standard of charges for the government of medical practitioners has been adopted, and it is considered a matter of principle and of honor to adhere to it as nearly as the varying circumstances of the people whom they serve will permit. These fee tables are always respected by the more honest and gentlemanly portion of the profession, whether members of a society or not. But it is a humiliating and lamentable fact, that the medical profession seems to be afflicted to a greater extent, in some communities, at least, than any other, with parasites, who do not reach, or even approximate, the standard of honorable men; and these usually succeed in "reaping the golden harvest," at the expense of the better bred and educated, who cannot sacrifice, or even compromise, their manhood on the altar of mammon. Not only do the dishonorable class alluded to bend the "supple hinges of the knee that thrift may follow fawning," but they resort, in some instances, to more reprehensible conduct to increase the list of their patients. Thus, by exaggerated statements of the number of their cases, and by an assumed devotion to study "that may be seen of men," they often succeed in gulling the unsuspecting public into a belief of their superior skill and professional attainments, ignoring the maxim that "real merit is always modest." It is a lamentable fact, that sometimes, otherwise intelligent men, and women, too, are more easily imposed upon by charlatans than any other class of pretenders. A fondness for novelty, and a leaning toward delusions and the unreal, probably is the explanation why a foothold for the vagaries of Hahnemann is still to be seen in our cities and towns. Educated men have left the ranks of legitimate medicine and espoused the name, at least, of the founder of the "do-nothing" calling; and for what? It is charitable to suppose, from mental aberration. But what should be done with the dishonorable still remaining within our own ranks, who, knowing the gullible side of human nature, take advantage of it in the manner alluded to above? The Law Association of Baltimore would doubtless reply, if asked of some of them, "expel them from the societies of which they are members, first; and should they still persist in their course, prosecute them." See article on this subject in February issue of this journal, under editorial head.

